# How Do Family Caregivers of Older People Give Up Caregiving?

**Published:** 2015-07

**Authors:** Hamed Mortazavi, Hamid Peyrovi, Soodabeh Joolaee

**Affiliations:** 1 Department of Geriatric Nursing, School of Nursing and Midwifery, North Khorasan University of Medical Sciences, Geronotological Care Center, Bojnurd, Iran;; 2 Nursing Care Research Center, Department of Critical Care Nursing, School of Nursing and Midwifery, Iran University of Medical Sciences, Tehran, Iran;; 3 Nursing Care Research Center, Department of Grontological Nursing, School of Nursing and Midwifery, Iran University of Medical Sciences, Tehran, Iran

**Keywords:** Aged, Home care, Family caregiver, Nursing home

## Abstract

**Background:**

Population aging has social, economic and political consequences. Most family caregivers prefer to care for their family member older person with chronic disease at home. Despite traditional culture within Iranian families, in some cases, hospitalization of the elderly in nursing home is inevitable, and this affects the old person and his/her family. The aim of this study was to explain how Iranian family cargivers give up caring their older person with chronic condition at home.

**Methods:**

A grounded theory approach was used to conduct the study. The study setting included four nursing homes under the auspices of Iran Welfare Organization. Fourteen participants were recruited through purposive sampling. Data were collected from December 2010 to March 2011 by Semi-structured interviews lasting about 17 to 95 minutes (average 52 minutes). Constant comparative analysis was used to analyze the data.

**Results:**

Three main categories appeared at the end of the analysis: “going out of the road of usual life”, “challenge of meeting older person, family and caregivers care needs”, and “the appearance of inconstancy in the family”. They explained exclusively how family caregivers of old people give up caregiving.

**Conclusion:**

Health care providers are recommended to become familiar with challenges of family caregivers in taking care of older person with chronic disease at home, and then organize their supportive and consulting actions according to family situations in order to improve the life quality of older person and family caregivers.

## Introduction

Worldwide population is aging rapidly due to success in controlling childhood diseases and maternal mortality and helping women achieve control over their own fertility.^1^ Population aging will result in social, economic and political consequences for families, social workers and the society.^2^ Dependency in daily life activities, sickness and previous experience of living in nursing home are predictive elements for old people’s transition to nursing home in America.^3^ The old people who suffer from chronic diseases are often dependent on others functionally and need long time caring.^4^ Diseases like stroke, diabetes and movement problems increase the risk of old people’s transfer to nursing home by 50 percent.^5^ Also, necessity to provide more skilled care, caregiver’s health status, behaviors resulting from dementia in patients, and the need for getting help from others for taking care of the old person were reported as the reasons for older people’s transition to nursing home.^6^

Most family caregivers participate in decision making about taking care of older person^7^ and prefer to care for the old family member  with chronic disease at home.^8^ However, families who try to provide financial, emotional and physical support for old family member find this support as a challenging experience.^2^ Despite traditional culture of Iranian families, in some cases, older person’s institutionalization in nursing home is inevitable, and this affects older person and his/her family.^9^

Iran, a developing country with more than 75 million people in the Middle East,^10^ has experienced significant changes in cultural, socioeconomic and demographic dimensions in recent decades, resulting in the growth of the elderly population.^11^ In Iran, the elderly population has increased from 3.9 percent in 1956 to 7.3 percent in 2006, and it is estimated to reach 10.7 percent in 2021. Over the last century, the number of old population shows a seven-fold increase.^12^

Iranian society, like other eastern societies, is a family-centered society, having kept its traditional beliefs about elder family members. According to these beliefs, care of the elderly is a sacred duty based on which most Iranians are willing to support and care their elder family member at home.^13^ Although the culture of placing elder people in nursing homes has not become a norm in Iran,^14^ statistics suggest incremental trend of placing the elderly in nursing homes among Iranian families.^15^ There is room for more scientific studies as well as development of older people care institutions to provide enough resources to face this issue.^16^

Limited researches have been conducted about problems of older people caregiving at home. Studies have shown that some older people have been transferred to nursing home voluntarily and some by obligation.^17^ Another research reported that most family caregivers experience fatigue and incresing responsibility during the caring of older people at home.^18^ Other studies have also indicated that some family caregivers encountered unsuitable care condition, lack of systems offering health services, and lack of formal support.^19^ No study has been conducted about the process of family caregivers’ decision making for transferring the old person with chronic disease to nursing home in Iran. This study reports how Iranian family cargivers give up the home care of older person with chronic condition. This report is a part of a larger study exploring how Iranian family cargivers decide to transfer their older family member with chronic disease to nursing home.

## Materials and Method

This study is a part of the first author’s doctoral thesis conducted to explain the process of Iranian family caregivers’ decision making about transferring older person with chronic disease to nursing home. Grounded theory approach was used to conduct the study, because the process of family caregivers’ decision making for the transition of elder person with chronic disease to nursing home is subject to socio-cultural context and various factors interact with this process. Grounded theory is the most appropriate method to describe and understand the structures of social and psychological processes in the social world.^20^

The study setting included four nursing homes under support of Iran Welfare Organization which provided the researchers’ access to informed participants with maximum variety, allowing them to receive a comprehensive description about Iranian family caregivers’ experiences of ending the care of older person with chronic disease at home.

Eleven family members, taking care of an old person with chronic disease before transition to nursing home, were recruited through purposive sampling based on inclusion criteria as follows: 1) having participated in decision making for transition of the elder family member with chronic disease to nursing home during the last six months, 2) being at least 18 years old, 3) being able to speak in Persian fluently, 4) being the relative of the old person by blood or marriage, and 5) not being affected by cognitive impairments.

After conducting some interviews and analyzing them, the researchers decided to interview with some old people with chronic disease living in nursing homes and also family caregivers having older person with chronic disease at home, because the collected data up to that time reflected some ambiguities requiring more exploration of the phenomenon from the viewpoints of the old people being cared for at home or in nursing home. Accordingly, the main researcher (HM) interviewed with two elderly person and one family member caregiver who was continuing caregiving at home.

Fourteen qualified key informants who had desired to participate in the study were informed about the study objectives. They completed and signed the written informed consent. Demographic information of the participants is shown in Table 1.

**Table 1:** Characteristics of participants and their respective old person cared for by them

Characteristics of participants (n=14)	
Gender, n (%)	
Male	8 (57)
Female	6 (43)
Age in years	
Mean, range	49, 40-78
Relation with elder person, n (%)	
Son	6 42.96)
Daughter	2 (14.26)
Son-in-law	1 (7.13)
Wife	1 (7.13)
Sister	1 (7.13)
Brother	1 (7.13)
Elder person	2 (14.26)
Economic status, n (%)	
Weak	6 (42.85)
Moderate	5 (35.71)
Good	3 (21.42)
Education, n (%)	
high school diploma	6 (42.96)
Graduate	8 (57.04)
Duration of caregiving (month)	
Mean, range	42, 4-180
Characteristics of elder people cared for by participants (n=14)	
Gender, n (%)	
Male	6 (42.96)
Female	8 (57.04)
Age in years	
Mean, range	79, 65-85
Living status before residence in nursing home, n (%)	
Alone	42.96 (6)
With family members	57.04 (8)
Duration of residence in nursing home (month)	
Mean, range	9, 1-17

After the research proposal was approved by “Ethics Committee in Medical Researches” of Tehran University of Medical Sciences, all potential participants were informed that participation would be voluntary. They were given oral and written information about the study aim, the interview process, and risks and benefits of the project. Those who agreed to be interviewed were informed that their words would be audio taped and transcribed by the researcher for analysis. They signed a written informed consent form. Participants were informed that they could terminate their participation whenever they wanted. Only the main researcher was aware of the real identities with their respective tapes, report or description. 

Data were collected from December 2010 to March 2011. Semi-structured interviews were conducted in Persian language, in a calm atmosphere convenient for participants in terms of time and place. Interviews started with the question “How did you decide to transfer your old family member to nursing home?” followed by probing questions such as “Is it possible to explain more?”, “Then, what did you do?” “When you say…, what do you mean?” The interviews were audio taped and then transcribed verbatim. The duration of interviews ranged between 17 to 95 minutes (average 52 minutes) according to the participants’ condition. During and immediately after each interview, the researcher made a note of important discussed points. Sampling continued until data saturation.

Constant comparative analysis suggested by Strauss and Corbin (1998) was used to analyze the data.^21^ In the first phase, open coded, the transcribed interviews were read several times to get the sense of the whole; then, the main words and phrases were highlighted to find substantive and implicit codes. During this stage, 3345 initial codes emerged. Next, based on constant comparison, the codes were compared together in terms of similarities and differences to classify under categories. Modifications were made repeatedly to develop categories and subcategories. In the second phase, axial coding, the level of abstraction of categories was improved. Moreover, the logical relations were drawn and the main category, causal conditions, context, actions/interactions strategies, consequences and intervening conditions were identified. Finally, in the third phase, the categories became more integrated and saturated, and the core variable was explored. To conduct comparisons, researchers applied personal recognition, professional knowledge, specialized texts and writings. Constant comparative analysis was implemented actively, providing a basis for theoretical sensitivity. Moreover, during constant comparisons, researchers were aware of ideas coming to the mind and immediately took a note in the form of memos. These memos helped researchers to know where they were, where they are now and where they should go.^21^

Doing constant comparative analysis, allocating adequate time to collect data, writing field notes and long contact with participants helped the researcher to reach a deep understanding of the participants’ experiences, thereby ensuring credibility. Also, member check was considered after the interviews were transcribed and following data analysis. During the analysis, the codes, categories, sub-categories, memos, diagrams and storyline were reviewed by expert professionals and two nursing doctoral students and their feedback was considered. After credibility was assured, dependability would be achieved.

Regarding confirmability, it was tried to write all research activities (audit trail) as other researchers can follow up what happened during the study. Regarding transferability, the researchers provided a thick description of the findings, helping the readers to judge how the findings could be transferable to different contexts. 

## Results

Three main categories appeared at the end of analysis: “going out of the road of usual life”, “challenge of meeting older person, family and caregivers care needs”, “the appearance of inconstancy in the family”.

*Going Out of the Road of Usual Life*

*Need for Constant Attendance*

Taking care of an old person with chronic disease at home requires constant attendance of one of the caregivers. In this regard, one daughter who was responsible for taking care of her old parents and had placed her father in nursing home by force, stated: “*anyway one must be at home; always one should be there. Children or I [must be] at home …*” (P#9). Also, one old person’s son said: “*My mother couldn’t see. She couldn’t recognize anybody and many times her legs and arms were injured, because of falling down and … we always were concerned about her …”* (p#4). One old person’s daughter stated: “*We couldn’t go anywhere, [for example] to a party …*” (p#3).

*Anarchy in Daily Life of Caregivers*

Most of family caregivers, after accepting the role of taking care of an old family member with chronic disease at home experienced anarchy in their daily life. One old person’s son said: “*Families have their own difficulties … my sisters left her home and long nights went and slept near mother and couldn’t train their children and … they cared for children less than before … my mother called her daughter, so she was at work and it made my sister experience stress to leave her work because my mother was alone at home …*” (p#2).

*The Challenge of Meeting the Elderly, Family and Caregivers’ Needs*

*Feeling Hopelessness after Seeking Supportive Resources*

Most family caregivers have adopted different resources to be able to take care of the elderly member of the family as well as do their own life activities. One of these supportive resources was the cooperation of other family members to take care of the old person in order to increase their caregiving power and provide a suitable opportunity to manage their life affairs. Some caregivers stated that because of taking care of the old person lonely, they had been forced to leave their work and life, so they didn’t have any choice but placing the old person in nursing home. One old person’s son said: “*Finally, I called my brother living in another city and told him this is a reality, my wife has left me. My life is lost and I am responsible for my famiy and I can’t go to work any longer. If I cannot work there would be no income …” *(p#6). Another old person’s son stated: “*…well, we reached a deadlock and I stated that either my mother should be at our homes or I declare that I am at the end and…”* (p#2).

One of helping resources for caring the old person with chronic disease at home was recruiting private nurse or receiving services from health organizations at home, but most family caregivers emphasized family members’ participation because they had found it preferable to seek help from out of the family. 

*Challenges of Providing an Appropriate Environment for Caregiving*

Most family caregivers expressed providing a suitable physical environment to take care of the old person with chronic disease as one of their concerns. One daughter said: “*… taking care of an old person in an 80 or 90 square meters apartment is very difficult. What is the capacity of this house; I have three daughters and two sons-in-low and grandchildren, and one room is exclusively allocated to him*” (p#3). One son said: “*For keeping the home clean, I wake her up at least three times since 12 midnight to 6 a.m. Whether she needed to go to restrooms or not, I took her there…I covered the floor by washable floorings so that if my mother makes it dirty, I can wash them. I put one bed near her and slept there…*” (p#1). One son taking care of his mother at home reported: “*I prepared one plastic mattress that doesn’t get dirty and finally whatever we do, home becomes dirty and smelly, but we attempt to tell nothing in order no to make her annoyed …*” (p#14).

*Old Person’s Costly Caregiving*

Most family caregivers indicated that taking care of an old person with chronic disease at home increased their living costs. This was obvious in cases of the elderly who didn’t have independent financial resources such as salary, pension or retirement payments, because the main caregiver must endure especial additional financial burden of treatment and the old person’s caring, too. One son stated: “*Every other day, we should pay 120-150 thousand Rials (12-15 dollars) for dressing my mother’s wounds. Maybe one has financial ability and employs one educated nurse, but many people don’t have enough money*” (p#1). Another son said: “*I made a phone call to private nursing institutes; they came and inserted a urinary catheter for my father with a bag attached and took 120 thousand Rials (12 dollars)…*” (p#6). One older person’s brother said: “*Actually, his treatment costs are very high, you know visiting physician has its own expenses … most doctors prescribe medications that are expensive and so it is difficult…” *(p#14).

*Being Caregivers’ Health at Risk*

Most family caregivers stated that because of taking care of older person and not having enough time to do their different roles, they have to ignore their own health so they experienced the feeling of physical and mental tiredness, and decreased energy and were involved in physical disorders. An old person’s spouse said: “*I have heart disease, whenever I take him to bathroom, I must suffer some difficulties and my situation becomes worse …*” (p#7). An old person’s son reported: “*During the last* *four months that I took care of my mother, I have been feeling ill. I was really affected with all diseases during this period, I had skin allergy and I had fungal disease*” (p#1). One old person’s daughter said: “*I myself have back and neck arthritis and I feel our life is breaking up and we are depressed. I have not gone out for about twenty days*” (p#3).

*Emerging Instability within the Family*

*Intensification of the Old Person’s Unsuitable Situation*

Most family caregivers reported that tolerating older person’s urinary and feces incontinency was hard for them and their families. One the old person’s son said: “*My mother had lost her normal condition and she couldn’t state her toilet and this was the only reason for placing her in nursing home … our life became dirty…*” (p#1). Another son stated: “*Many times I couldn’t stand, because he made himself dirty, in a small house; then my son came and kept his nose. I became nervous and suddenly shouted and … I said it is a reality, why you keep your nose … he is not blameworthy … well, the home is dirty … taking care of him was very hard for us*” (p#10).

*Contradiction between Family Members and Old Person’s Interests or Benefits*

Some family caregivers stated that their children couldn’t tolerate their absence that results from constant caring of their grandmother. One of the sons said: “*Children complained and said: doesn’t grandma have other children! Why you take care of her. You took my mother and father; we don’t have mother and father. My daughter, who is at pre-university stage, came and shouted in front of all family that we didn’t see our parents for three months. She came to grandfather’s home and said I come to see my father … Children have become weary …*” (p#1). Some family caregivers indicated that the cultural difference between the older person and grandchildren about using media had produced some challenges among family members. One older person’s son taking care of her mother at home said: “*Children like to be updated; they like to watch TV loudly. She is an old person and says: turn it down or what program do you watch!, change it, she is seeking excuses, but our children are instructed and respect her …*” (p#14). Some family caregivers stated they were in a situation that they had to choose between the old person and their children. An old person’s sister said: “*My brother’s children grumbled and were unsatisfied about my brother’s presence in their home. My brother couldn’t disregard them …*” (p#8). Another old person’s son said: “*We compromised our mother for 15 years. We are at the end and her status is going to become worse and her expectations will increase. It is her right. She is ill, she is not healthy, but it can’t be a reason of breaking up my life. I can’t force my wife to agree with my mother … it is not just me, so all of my family*” (p#2).

*Reaching a Deadlock in Caregiving*

After reaching a deadlock to continue caring at home, most family caregivers didn’t have any choice but sending the old person to a nursing home. An old person’s daughter said: “*We really discussed to accept sending her to a nursing home … we were struggling about this decision for a few years. We attempted enough but we can’t continue … decision making was a strange situation for us…*” (p#3). An old person’s son said: “*Finally, I phoned my brother who lived in another city and I said … my wife has left me. My life was broken and I can’t go  to work … for three months it was my duty that I couldn’t work, even one hour I couldn’t leave him.*” (p#6). Another son stated: “*It was a stage at which we couldn’t control the situation…” *(p#10).

In some cases, the old person reaches a deadlock for being at home and he/she wants to be sent to a nursing home. A woman residing in nursing home reported: “*After six years that I had stroke and was at home, I said to myself that I want to go to nursing home. My brother and others said no, I said if you don’t do it, I myself go. I am tired and they too and …*” (p#8).

## Discussion

Our study focused on Iranian family caregivers with an old person resident in nursing home; we aimed to find out how they give up taking care of the old person with chronic disease at home. Another limitation is that participants had suffered such situation long before taking part in this study. The explanatory report of their decision making experience was subject to recall bias.

This study was the first one in Iran to conceptualize how family caregivers of an old person with chronic disease give up caregiving at home. 

According to the findings, family caregivers had accepted taking care of the elderly family member because of disability of the older people in doing their personal activities and fear of damaging themselves and others. Also, a predominant social expectation was another reason for accepting the role of the old person’s caring at home by family caregivers. 

Findings showed that taking care of an old person at home requires a relatively full time presence of one family member, and this leads to persistent concern for caregivers. Studies have shown that most of the family caregivers had constant anxiety because of day and night taking care of their old person and didn’t have mental peace.^9^ In another study, most family caregivers had experienced problems in social relations with others because of accepting caregiver roles.^18^

In the present study like the others, most family caregivers after accepting the role of caring were forced to attend permanently to take care of the old person, so it caused daily life disorders. Studies have shown that taking care of an old person affects family caregivers’ performance.^4^ another researcher reported that family caregivers had experienced disturbance in life quality and family relations after accepting this responsibility.^22^

Our findings indicated that all family caregivers were seeking other members’ cooperation to continue home caring and usual life process, and because other family members didn’t cooperate, their caring intention had decreased gradually. The findings of other studies showed that changes in behavioral patterns, health and old person’s caring needs caused a decline in caregivers’ energy and they requested other members’ help and systems that provided official services; and if they didn’t support, they felt lonely in caring difficulties and didn’t have any choice but sending their old person to a nursing home.^4^^,^^19^^,^^22^^,^^23^ Contrary to our finding, another researcher reported that in spite of enough centers providing health services for the elderly at home, most family caregivers had fewer tendencies to hospitalize the old person in official centers in spite of the old person’s dangerous health status.^24^ It seems that what distinguishes our finding from other studies is the focus on lack of cooperation from other family members to take care of the old person at home because official supportive resources don’t consider any plans to support family caregivers who have an elderly with chronic disease at home.

According to the findings, family caregivers were seeking the choice of private nurse employment after disappointing from other members’ cooperation in caregiving, so that they can be helped by them for a long time. Another researcher reported that family caregivers had delayed their decision to place their old family member in nursing home by using supportive programs for home caring or private nurse employment.^25^ In another study, family caregivers looked for help in to take care of the old person by employing a private nurse.^22^ It seems that what differentiates our findings from others was the lack of nurses with university degree in Iran and inadequate centers providing rehabilitation and caring services for the old people. So, family caregivers for getting assistance in doing caring needs, were forced to employ a worker and this choice had many challenges such as the old person’s disagreement with that worker because of cultural differences, not accepting the oldperson’s situation by the caregiver because of worst health and low quality of provided services and these facts had caused many challenges in continuing home caring for family caregivers.

According to our study findings, most family caregivers had problems in providing a suitable situation for caring the elderly at home because of limitations in physical environment and need to prepare the home atmosphere; on the other hand, caregivers being tenant increased these caring challenges at home for arrangements in the house. The study findings showed that by changing older person caring needs at home family caregivers were trying to improve the home environment for caring; all these changes were done to make the caring easy so that home situation becomes a safe place for the old person with dementia.^26^

Our study findings showed that accepting the role of taking care of an old person with chronic disease at home increased life expenses, and they didn’t predict them. The transportation costs of the old person for needed treatment activities, the rise of diagnosis and treatment costs, providing required drugs, providing adult diapers and high costs of consumer goods for the old person were some of these costs. Other study findings indicated that family caregivers should pay additional caring costs of their old person in addition to their life and family expenditures and without non-official support or social-financial resources, apparently it leads to sending the old person’s entrance to nursing home.^3^^,^^4^^,^^25^^,^^27^^,^^28^ In Iran, paying costs of taking care of an old person is the duty of family caregivers and the government doesn’t have any obligation to provide these costs. These factors along with inadequate coverage of insurances providing health services cause family caregivers to experience more challenges. So, holding a comprehensive program to support caregivers with an old person at home by government, such as planning for the old person’s insurance or paying subsides to family caregivers can decrease family concerns.

In the current study, family caregivers had experienced physical and mental problems during taking care of the old person with chronic disease at home, similar to the finding that have been reported by other studies.^19^^,^^27^^,^^29^ In addition, our study findings showed that some family caregivers couldn’t take care of the elderly person because they had chronic disease and their health was in danger and they couldn’t continue home caring. Another researcher reported that family caregivers who had at least one chronic disease attributed the worst symptoms of their disease to hard caring role of the old family member and decided to transfer the old person in nursing home.^22^

According to the findings, family caregivers had experienced serious challenges to take care of the old person at home because of the old person’s worst health status especially urine and fecal incontinency. In some cases, these challenges reached a point that made home caring impossible. Previous study findings showed that family caregivers expressed concern about deteriorating an old person’s health and about the problems of controlling the old person’s behaviors resulted from chronic disease.^6^^,^^22^^,^^26^^,^^27^ The study findings indicated that delay in nursing home placement can provide great savings in systems providing health services. Anyway, savings in health system costs related to delay in nursing home placement need to balance in social level by spending them for family caregivers in order to provide unpaid unofficial caregiving.^29^ When the health status of the old person deteriorates, caregivers feel helpless in taking care. This situation exaggerates the condition. On the other hand, most nursing homes in Iran have just caring facilities and refuse to take care of the old person with chronic disease in critical situation that this reality increases the caregivers’ difficulties. So, by arranging supportive programs available at home, it seems that supporting these caregivers in critical situations can relieve their burden.

Our findings showed that old people with chronic situation, their expectations and inevitable struggle with them were very hard for family caregivers. Studies have shown that some family caregivers stated that they were forced to struggle with their challenging relations; they refused to eat, use drugs and wash their teeth, because they felt their private life has  been attacked and also encountered with disastrous situation.^26^ Other study findings showed that when family caregivers need to rest, the old person needs to be cared and when the old person is in rest, the caregiver should do daily life activities and this incongruence was a disappointing and hard experience.^27^

According to the current study findings, family caregivers stated that when they were taking care of the old person at home, there was a contrast between the old person and members profits; in some cases, caregivers were in situations that they didn’t know whether they continue home caring and disagree with other members’ wills or pay attention to their wills and ignore the old person’s wishes. Finally, this confusion caused them to give up caring the old person at home and seek a remedy to transfer a suitable alternative for caring the old person. Studies have shown that family caregivers have some problems for making a balance between their responsibilities for the old person and the growth of their family members such as education or marriage, so they experience moral dilemma.^4^

The findings showed that after accepting the caring role, family caregivers experienced disturbance in their usual life process by constant presence for caring the old person with chronic disease, and encountered challenges to balance their responsibilities to meet the needs of the old person, themselves and family members, and this caused inconstancy in the family. Finally, family member caregivers had no choice but to send the old person to a nursing home after reaching a deadlock. Another study reported that family caregivers considered the reasons for transferring the old person to a nursing home as an inevitable and due to reaching a limitation.^27^ Another study stated that most family caregivers were exhausted because of long term caregiving.^8^ Similarly, another study reported that family caregivers stated that the feeling of having no other choice but putting the old family member in a nursing home, the sense of less support, and tendency toward governmental support are the factors that made the caregiver uncontrolled.^22^ One study reported that when family caregivers felt unable in taking care of their old person at home, they were seeking some institutions from out of family and didn’t have any choice but sending the old person to a nursing home.^9^ Also, family caregivers reached a deadlock in taking care of the elderly person because of accepting the caring alone and losing their jobs; they were forced to decide to send their old person in nursing home. Similarly, another study reported that family caregivers stated that they should leave their work because of taking care of the old family member.^4^

## Conclusion

Most family caregivers encounter serious challenges in taking the role of caregiving for the old person with chronic disease at home. What distinguish Iran from other countries is the cultural values of caregiving the old person at home by people. Therefore, the government not only doesn’t consider a comprehensive program to support family caregivers in order to continue old person’s caring at home, but also doesn’t recognize its challenges.

Health care providers are recommended to become familiar with challenges of family caregivers in taking care of an old person with chronic disease at home, and to organize their supportive and consultation actions according to family situations in order to improve the life quality of the old people and family caregivers. 
